# Human Ultrasound Neuromodulation: State of the Art

**DOI:** 10.3390/brainsci12020208

**Published:** 2022-02-01

**Authors:** Roland Beisteiner

**Affiliations:** Department of Neurology, Functional Brain Diagnostics and Therapy, High Field MR Center, Medical University of Vienna, Spitalgasse 23, 1090 Vienna, Austria; roland.beisteiner@meduniwien.ac.at

The first human applications of ultrasound in medicine date back to 1939, when Reimar Pohlmann (Berlin, Germany) published data on therapy of neuralgia with ultrasound [1]. The first diagnostic ultrasound application was developed by Karl Dussik (Vienna, Austria), who created a projection image of the brain, based on differences in ultrasound absorption by different brain components [2]. In 1958, the Fry brothers published the first demonstrations of neuromodulation with focused ultrasound in animals [3]. Despite ultrasound in medicine starting with neurological applications, the history of human ultrasound neuromodulation is rather short, but with a tremendous recent uprise. A milestone has been achieved by the development of techniques to focus ultrasound through the intact human skull [4]. Important insights for the development of human and medical applications also came from animal studies [5]. According to Fomenko et al. [6], the first human studies were then published from 2013, starting with a diagnostic and unfocused ultrasound system [7]. The authors report that ultrasound neuromodulation in subjects suffering from chronic pain reduced their pain perception and improved their global affect. The first human neuromodulation studies using a *focused* ultrasound technology were published in 2014 [8,9]. In their study in healthy subjects, Legon et al. [8] showed precise targeting of the primary somatosensory cortex within millimeter range. The authors describe the successful neuromodulation of electroencephalographic signals (EEG) and improved sensory discrimination. In 2014 as well, the first clinical study was published [9] with a non-navigated but focused transcranial ultrasound technology based on ultrashort pulses. In patients with unresponsive wakefulness syndrome, long-term application of ultrasound neuromodulation improved the patients’ ratings on various clinical coma scales. This was followed by a case report on the improvement from minimally conscious state with navigated and focused stimulation of the right thalamus [10]. The first clinical study in a group of patients with navigated focused ultrasound was published in 2019 [11]. Their newly developed Transcranial Pulse Stimulation (TPS) technique targeted the memory network nodes in 35 Alzheimer patients. Authors reported a significant cognitive improvement of their patients over a period of 3 months and a connectivity improvement of the memory network, documented by fMRI. Other pioneering patient investigations exist for depression [12], epilepsy [13] and Parkinson’s (performed with an unfocused diagnostic system, [14]). The first structural changes induced by human ultrasound neuromodulation have also been published recently using the TPS technology [15,16]. With more than 60 clinical and research centers established, TPS, meanwhile, is the most widely used technology for human ultrasound neuromodulation.

Compared to electromagnetic neuromodulation technologies such as Transcranial Magnetic Stimulation (TMS) or Transcranial Direct Current Stimulation (TDCS), ultrasound-based techniques possess two distinct advantages: (1) the targeting of stimulation foci does not depend on the intracerebral conductivity situation—which may grossly be changed due to brain pathology—and (2) ultrasound is not limited to cortical stimulation but is the first non-invasive technique which may precisely target deep areas of the human brain. Although much methodological research concerning stimulation approaches has yet to be conducted [17], human ultrasound neuromodulation provides completely novel options for neuroscientific research, including brain stem targets [18] and clinical therapy [19].

*Brain Sciences* has now taken an editorial lead on this revolutionary topic and is starting a paper collection to give a comprehensive overview of the state of the art and prospective potential of these technologies (https://www.mdpi.com/journal/brainsci/special_issues/Neuromodulation_System). The collection will feature timely insights in methodological issues, cellular mechanisms, basic research, neuroscientific potential, translational issues and clinical applications. It will also provide outlooks towards future neuroscientific and clinical developments. The collection starts in this issue of *Brain Sciences* with an article on focused ultrasound as a translational tool by NM Spivak, JL Sanguinetti and MM Monti.

## References

[1] Pohlmann R., Richter R., Parow E. (1939). About the spread and absorption of ultrasound in human tissue and its therapeutic effect on sciatica and plexus neuralgia. Dtsch. Med. Wochenschr..

[2] Dussik K.T. (1942). Über die Möglichkeit, hochfrequente mechanische Schwingungen als diagnostsches Hilfsmitel zu verwenden. Z Ges. Neurol. Psychiat..

[3] Fry F.J., Ades H.W., Fry W.J. (1958). Production of Reversible Changes in the Central Nervous System by Ultrasound. Science.

[4] Clement G., Hynynen K. (2002). A non-invasive method for focusing ultrasound through the human skull. Phys. Med. Biol..

[5] Leinenga G., Götz J. (2015). Scanning ultrasound removes amyloid-β and restores memory in an Alzheimer’s disease mouse model. Sci. Transl. Med..

[6] Fomenko A., Neudorfer C., Dallapiazza R.F., Kalia S.K., Lozano A. (2018). Low-intensity ultrasound neuromodulation: An overview of mechanisms and emerging human applications. Brain Stimul..

[7] Hameroff S., Trakas M., Duffield C., Annabi E., Gerace M.B., Boyle P., Lucas A., Amos Q., Buadu A., Badal J.J. (2013). Transcranial Ultrasound (TUS) Effects on Mental States: A Pilot Study. Brain Stimul..

[8] Legon W., Sato T.F., Opitz A., Mueller J., Barbour A., Williams A., Tyler W.J. (2014). Transcranial focused ultrasound modulates the activity of primary somatosensory cortex in humans. Nat. Neurosci..

[9] Lohse-Busch H., Reime U., Falland R. (2014). Symptomatic treatment of unresponsive wakefulness syndrome with transcranially focused extracorporeal shock waves. NeuroRehabilitation.

[10] Monti M.M., Schnakers C., Korb A.S., Bystritsky A., Vespa P.M. (2016). Non-Invasive Ultrasonic Thalamic Stimulation in Disorders of Consciousness after Severe Brain Injury: AFirst-in-Man Report. Brain Stimul..

[11] Beisteiner R., Matt E., Fan C., Baldysiak H., Schönfeld M., Novak T.P., Amini A., Aslan T., Reinecke R., Lehrner J. (2020). Transcranial Pulse Stimulation with Ultrasound in Alzheimer’s Disease—A New Navigated Focal Brain Therapy. Adv. Sci..

[12] Reznik S.J., Sanguinetti J.L., Tyler W.J., Daft C., Allen J.J. (2020). A double-blind pilot study of transcranial ultrasound (TUS) as a five-day intervention: TUS mitigates worry among depressed participants. Neurol. Psychiatry Brain Res..

[13] Stern J.M., Spivak N.M., Becerra S.A., Kuhn T.P., Korb A.S., Kronemyer D., Khanlou N., Reyes S.D., Monti M.M., Schnakers C. (2021). Safety of focused ultrasound neuromodulation in humans with temporal lobe epilepsy. Brain Stimul..

[14] Nicodemus N.E., Becerra S., Kuhn T.P., Packham H.R., Duncan J., Mahdavi K., Iovine J., Kesari S., Pereles S., Whitney M. (2019). Focused transcranial ultrasound for treatment of neurodegenerative dementia. Alzheimer’s Dementia Transl. Res. Clin. Interv..

[15] Popescu T., Pernet C., Beisteiner R. (2021). Transcranial ultrasound pulse stimulation reduces cortical atrophy in Alzheimer’s patients: A follow-up study. Alzheimer’s Dementia Transl. Res. Clin. Interv..

[16] Matt E., Kaindl L., Tenk S., Egger A., Kolarova T., Karahasanović N., Amini A., Arslan A., Sariçiçek K., Weber A. (2022). First evidence of long-term effects of transcranial pulse stimulation (TPS) on the human brain. J. Transl. Med..

[17] Fomenko A., Chen K.-H.S., Nankoo J.-F., Saravanamuttu J., Wang Y., El-Baba M., Xia X., Seerala S.S., Hynynen K., Lozano A.M. (2020). Systematic examination of low-intensity ultrasound parameters on human motor cortex excitability and behavior. eLife.

[18] Guerra A., Vicenzini E., Cioffi E., Colella D., Cannavacciuolo A., Pozzi S., Caccia B., Paparella G., Di Stefano G., Berardelli A. (2021). Effects of Transcranial Ultrasound Stimulation on Trigeminal Blink Reflex Excitability. Brain Sci..

[19] Beisteiner R., Lozano A.M. (2020). Transcranial Ultrasound Innovations Ready for Broad Clinical Application. Adv. Sci..

